# Organization and Evolution of Primate Centromeric DNA from Whole-Genome Shotgun Sequence Data

**DOI:** 10.1371/journal.pcbi.0030181

**Published:** 2007-09-28

**Authors:** Can Alkan, Mario Ventura, Nicoletta Archidiacono, Mariano Rocchi, S. Cenk Sahinalp, Evan E Eichler

**Affiliations:** 1 Department of Genome Sciences, University of Washington School of Medicine, Seattle, Washington, United States of America; 2 Department of Genetics and Microbiology, University of Bari, Bari, Italy; 3 Department of Computing Science, Simon Fraser University, Burnaby, British Columbia, Canada; 4 Howard Hughes Medical Institute, Seattle, Washington, United States of America; The J. Craig Venter Institute, United States of America

## Abstract

The major DNA constituent of primate centromeres is alpha satellite DNA. As much as 2%–5% of sequence generated as part of primate genome sequencing projects consists of this material, which is fragmented or not assembled as part of published genome sequences due to its highly repetitive nature. Here, we develop computational methods to rapidly recover and categorize alpha-satellite sequences from previously uncharacterized whole-genome shotgun sequence data. We present an algorithm to computationally predict potential higher-order array structure based on paired-end sequence data and then experimentally validate its organization and distribution by experimental analyses. Using whole-genome shotgun data from the human, chimpanzee, and macaque genomes, we examine the phylogenetic relationship of these sequences and provide further support for a model for their evolution and mutation over the last 25 million years. Our results confirm fundamental differences in the dispersal and evolution of centromeric satellites in the Old World monkey and ape lineages of evolution.

## Author Summary

Centromeric DNA has been described as the last frontier of genomic sequencing; such regions are typically poorly assembled during the whole-genome shotgun sequence assembly process due to their repetitive complexity. This paper develops a computational algorithm to systematically extract data regarding primate centromeric DNA structure and organization from that ∼5% of sequence that is not included as part of standard genome sequence assemblies. Using this computational approach, we identify and reconstruct published human higher-order alpha satellite arrays and discover new families in human, chimpanzee, and Old World monkeys. Experimental validation confirms the utility of this computational approach to understanding the centromere organization of other nonhuman primates. An evolutionary analysis in diverse primate genomes supports fundamental differences in the structure and organization of centromere DNA between ape and Old World monkey lineages. The ability to extract meaningful biological data from random shotgun sequence data helps to fill an important void in large-scale sequencing of primate genomes, with implications for other genome sequencing projects.

## Introduction

**Figure 1:**
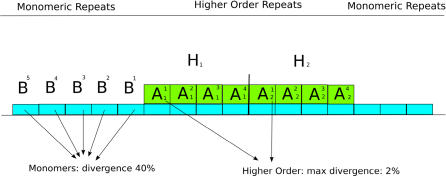
Composition of Human Centromeric DNA (A–B) Represented are ∼171-bp monomers: (A) in HOR; (B) in monomeric tracks. The divergence of the higher-order monomers marked with the same subscript is less than 2%.

Alpha-satellite is the only functional DNA sequence associated with all naturally occurring human centromeres. Alpha satellite consists of tandem repetitions of a 171-bp AT-rich sequence motif (called a *monomer*). In humans, two distinct forms of alpha-satellite are recognized based on their organization and sequence properties. In humans, a large fraction is arranged into higher-order repeat (HOR) arrays (also known as chromosome-specific arrays) where alpha-satellite monomers are organized as multimeric repeat units ranging in size from 3–5 Mb [1]. While individual human alpha satellite monomer units show 20%–40% single-nucleotide variation, the sequence divergence between higher-order repeat units is typically less than 2% [2,3] (Figure 1). The number of multimeric repeats within any centromere varies between different human individuals and, as such, is a source of considerable chromosome length polymorphism. Unequal crossover of satellite DNA between sister chromatid pairs or between homologous chromosomes during meiosis is largely responsible for copy-number differences and is thought to be fundamental in the evolution of these HOR arrays. The organization and unit of periodicity of these arrays are specific to each human chromosome [4,5], with the individual monomer units classified into one of five different suprafamilies based on their sequence properties [5,6]. Interestingly, studies of closely related primates, such as the chimpanzee and orangutan [2,7] indicate that these particular associations do not persist among the centromeres of homologous chromosome, implying that the structure and content of centromeric DNA changes very quickly over relatively short periods of evolutionary time.

In addition to higher-order arrays, large tracts of alpha-satellite DNA have more recently been described that are devoid of any HOR structure [6,8–11]. The individual repeats within these segments show extensive sequence divergence and have been classified as “monomeric” alpha-satellite DNA. Such monomeric tracts are frequently located at the periphery of centromeric DNA [9,11,12]. Consequently, unlike higher-order arrays, some of these regions have been accurately sequenced and assembled because they localize in the transition regions between euchromatin and heterochromatin. Phylogenetic and probabilistic analyses suggest that the higher-order alpha-satellite DNA emerged more recently and displaced existing monomeric repeat sequence as opposed to having arisen by unequal crossing-over of local monomeric DNA [8].

Centromeres and pericentromeric regions are frequently poorly assembled in primate whole-genome sequence assemblies [13–15]. These regions are generally regarded as too difficult to accurately sequence and assemble strictly from whole-genome shotgun (WGS) sequence. However, most WGS sequencing efforts include substantial amounts of alpha-satellite repeat sequence. Indeed, as much as 2%–5% of the sequence generated from the underlying WGS consists of centromeric satellite sequences—such data most often remain as unassembled in public database repositories.

In this study, we develop computational methods to systematically identify and classify alpha-satellite sequences from primate WGS sequence. We predict novel HOR structures from uncharacterized primate genomes and define the phylogenetic relationship of these sequences within the context of known human HOR satellite sequences. Finally, we take advantage of publicly available cloned resources to experimentally validate the dispersal of these newly described alpha-satellite sequences within various primate genomes. The data provide the first genome-wide sequence analysis of alpha-satellite DNA among primates from WGS data and a framework to identify and characterize more repeat-rich, complex regions of genomes as part of genome sequencing projects.

## Results

### Reconstructing HOR Repeat Structures

We took advantage of the extensive annotation of human centromeric DNA in the literature to initially construct a non-redundant database of HOR monomeric repeat sequences. We then retrieved WGS sequence data from four primate genomic libraries, identified alpha-satellite monomers using RepeatMasker, and extracted all alpha-satellite repeat units of ∼171 bp in length (Table 1). Our analysis indicated that approximately 1%–5% of all end-sequenced clones generated as part of the WGS libraries represented potential centromeric subclones. Although each library represents only 0.05–0.3 sequence coverage for each genome, human higher-order alpha-satellite arrays are typically 3–5 Mb in length, with hundreds to thousands of copies of each individual unit per chromosome. Consequently, each human HOR unit would be expected to be represented multiple times despite the relatively low coverage of the sequence library.

**Table 1:**
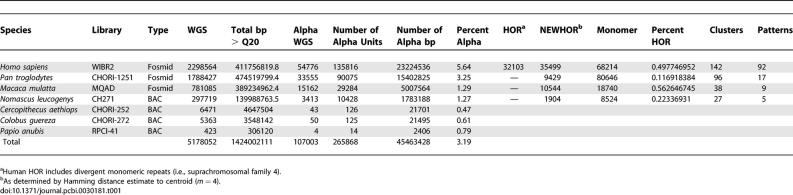
Genomic Library Sources and Alpha-Satellite Classification

We compared human WGS alpha-satellite sequences identified within the WIBR2 library to the non-redundant set of HOR sequences by pairwise alignment [16] and Hamming distance [17]. A total of 70% (132 of 188) of human HOR sequences were specifically identified within WGS sequence data (at most 4-bp mismatches), with an average representation of 240 reads per HOR monomer unit. We note that the representation of particular classes was variable and less than the expected number (*R* ^2^ = 0.13–0.09) as predicted by published minimum and maximum length of each array (Tables S1 and S4, Figure S1). In several cases (e.g., D8Z1, D9Z1, and D16Z1), sequence corresponding to the published HOR arrays was not discovered once within the library (Table 2). We repeated this analysis with additional sources of human WGS sequence and obtained similar results (Tables S1 and S4). The underrepresentation of particular sequences may indicate subcloning biases, variation in copy number, and/or sequence variation between centromeric HOR and published canonical alpha-satellite sequences.

**Table 2:**
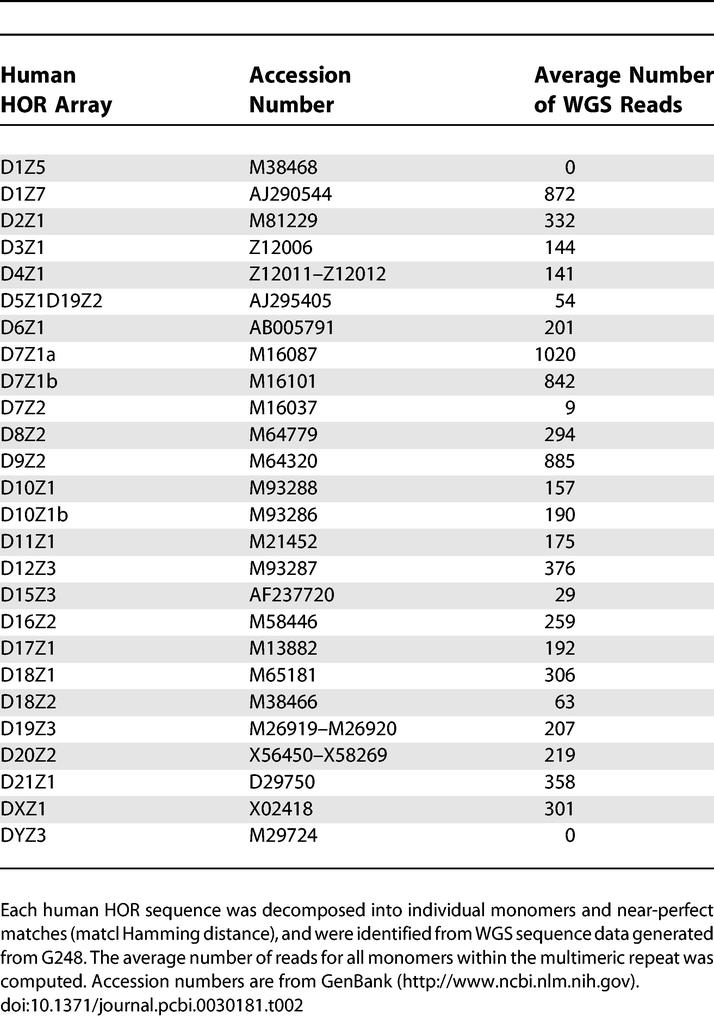
Representation of Known Human HOR Alpha-Satellites in WGS Libraries

We performed a pairwise analysis of all 135,816 human monomers retrieved from the human WIBR2 library (see Methods). Based on this self-comparison and the sequence similarity to published human HORs, we classified each monomer into one of three categories: (1) those that clustered with our dataset of published higher-order centromeric satellites; (2) those that clustered with each other but did not intersect those in (1); and (3) those that failed to cluster. Since our goal was to recover novel HOR sequences, clusters were established where all members showed at maximum 4-bp differences with any other member in a cluster. This target threshold was chosen because individual alpha-satellite sequences typically exhibit <2% sequence divergence with other paralogous members within a tandem array [18]. By these criteria, 23.3% (31,691 of 135,816) of the recovered monomers clustered with known HORs, with an equivalent proportion (26.2% or 35,499) grouping into 142 HOR clusters not apparently represented in our original dataset. The remaining 68,214 (50%) alpha-satellite monomers represent divergent HOR sequences or putative monomeric alpha-satellite lacking higher-order structure.

**Figure 2:**
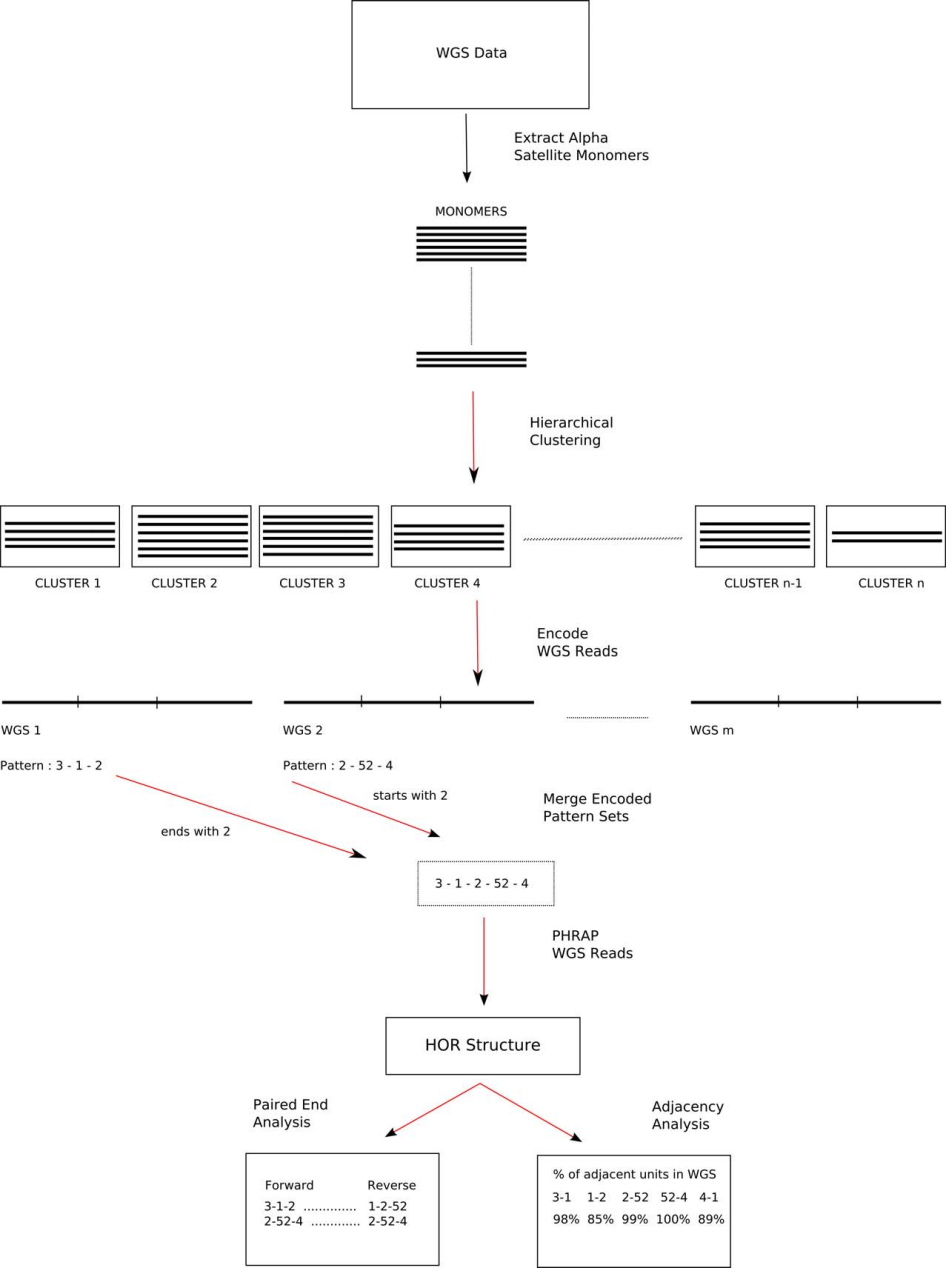
Flowchart of the *HORdetect* Algorithm Given a WGS sequence library, we first extracted alpha-satellite monomers from WGS sequence reads; performed hierarchical clustering to group highly similar monomers; encoded each WGS read with a unique cluster ID; and merged similar pattern sets. WGS sequences with the same encoded pattern set are assembled via PHRAP. The corresponding sequence (contig) is analyzed (paired-end and adjacency analysis).

WGS sequence reads corresponding to each cluster (type 2, as discussed above) were then retrieved, and each related sequence read was encoded based on its cluster composition (Figure 2). We would expect different monomeric units within different arrays to cluster if they are organized as HOR units. Based on the average read length, a typical WGS read should, then, consist of approximately three distinct HOR monomers. Encoded read compositions were then grouped into larger pattern sets based on a reiterative clustering algorithm. As expected, the pattern set ultimately looped as a result of tandem repetition of the array. We created sequence assemblies (PHRAP; default parameters, -forcelevel=10) [19,20] for all pattern sets that included 30 or more independent WGS sequence reads. A total of 18 distinct sequence contigs were created where the array length (*k*) ranged from 3–20 subunits.

Each assembled sequence contig was searched against GenBank (nr database) by BLAST (default parameters, *p = blastn*). We found that 3 of 18 patterns sets corresponded to higher-order alpha-satellite arrays, which had not been included in the original HOR set as part of our literature survey, while another 14 pattern sets showed sequence similarity to other human HOR but were discrepant with respect to published reports either in being more sequence divergent or incomplete with respect to the structure (e.g., D12Z3, D17Z1, D18Z1, etc). In the end, all but one computationally predicted HOR pattern set from the human WGS could be reconciled with published datasets (literature or GenBank).

**Figure 3:**
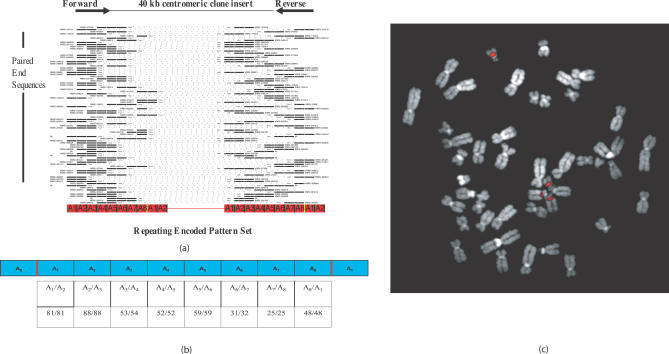
Novel HORs in Human (A) Paired-end sequence confirmation. Mate-pairs corresponding to a previously undescribed human pattern set (predicted 8-mer higher-order array) are shown. Black lines represent the left and right end sequences of each insert mapping to the same repeating encoded pattern set (red bars); dashed lines correspond to the unsequenced portion of the insert (40 kb in this case). The majority of end-sequence pairs map to the same repeat, confirming long-range tandem repeat organization. (B) Adjacency statistics for the new human higher-order array. The adjacency statistics simply calculates the number of times a specified monomer within the WGS sequence read maps adjacently to another specified monomer within the predicted HOR unit. The table shows data for a new human HOR sequence in Chromosomes 14 and 22. (C) FISH mapping (fosmid probe 3219L12) of predicted human higher-order array against metaphase spread of human chromosomes shows signals specific to Chromosomes 14 and 22 centromeres. Multiple clones from this encoded pattern set (3343N10, 3361F03, 3355D04, and 3355D08) showed identical results.

Our analysis predicted one potentially novel 8-mer HOR unit (HSAHOR8; Table 3) with 92% sequence similarity and 99% query coverage to a clone from Chromosome 22, and only 85% sequence similarity and 94% query coverage to published alpha-satellite sequence D2Z1 (Figure 3). In order to validate its structure, we performed a number of computational and experimental analyses. As a measure of homogeneity, we computed an adjacency statistic that simply calculates the number of times a specified monomer within the WGS sequence read maps adjacently to another specified monomer within the predicted HOR unit (Figure 2). If this repeat were organized as a multimeric tandem array, we would expect encoded monomers to map adjacently at a high frequency. This adjacency statistic for this novel HOR repeat ranged from 97%–100%, indicating considerable homogeneity in the organization of the repeat unit (Figure 3B).

**Table 3:**
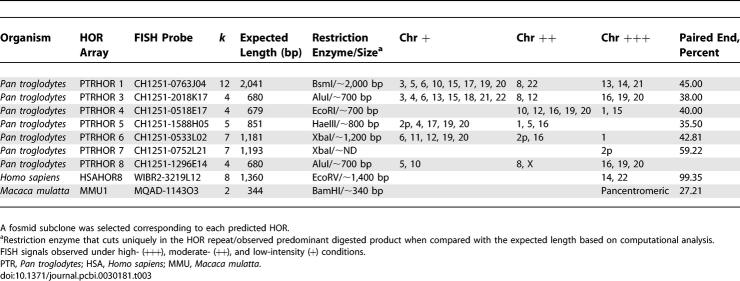
FISH and Restriction Digest Validation of Subcloned Primate Alpha-Satellite Sequences

Next, we analyzed mate–pair information associated with the WGS sequence reads. In our model, we would predict that HOR units should be repeated hundreds of times to form a large array of centromeric sequence typically several megabases in length. Consequently, corresponding end sequences from human fosmid clones should both map to the same encoded pattern set even though the two ends are separated by more than 40 kb. For 155 of 156 end-sequence pairs, we observed both the forward and reverse WGS sequences mapping to the same (encoded pattern set) or HOR unit, confirming long-range tandem repeat organization within the clone. As a final test, we performed fluorescence in situ hybridization (FISH) analyses using five different 40-kb fosmid clones representative of this new HOR array, using each as a probe in metaphase hybridizations (Figure 3C). FISH confirmed a typical centromeric HOR pattern, with signals observed on Chromosomes 14 and 22 (Figure 3C) for each of the five probes.

Our initial analysis was biased by triaging alpha-satellite sequences that clustered with known HOR units. As such, we favored accurate reconstruction of these by partitioning the sequence complexity. As a test of de novo alpha-satellite HOR reconstruction, we repeated our computational prediction of new higher-order arrays without excluding repeat units that map to HOR sequence (Table 4). In this blind test, we accurately predicted 12 of 24 known higher-order arrays with more than 92% sequence similarity. If we increase the maximum allowed Hamming distance from 4 to 6, we recover two more arrays with sequence identity greater than 92% (Table 4). This is likely a reflection of underrepresentation of particular classes of HOR sequence within WGS data (Table S1). Although not all classes of human HORs could be recovered, this analysis suggested that the approach could be implemented to discover a subset of previously undescribed HOR structures in uncharacterized genomes.

**Table 4:**
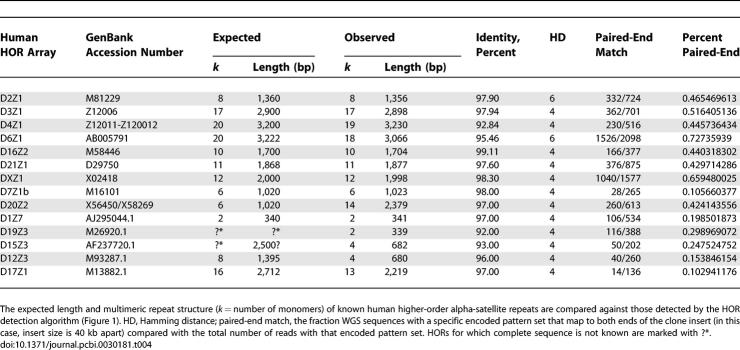
Reconstruction of Human Higher-Order Alpha-Satellite Repeat Structures

### Discovery of Novel Nonhuman Primate Alpha-Satellite DNA

In an effort to discover novel centromeric HOR units and to compare centromeric DNA in other primate genomes, we repeated our analysis for publicly available chimpanzee, gibbon, and macaque fosmid and bacterial artificial chromosome (BAC) end sequences. We extracted and classified all monomeric alpha-satellite DNA into two groups: monomeric (lacking HOR structure by our criteria) or HOR (evidence for HOR structure within WGS data) (Table 1) for each species. We identified encoded pattern sets in each species and assembled potential higher order repeats (Table 3). Upon analysis of macaque “higher-order” arrays, *all* potential multimeric repeat units collapsed into a core dimeric repeat structure (see Figure S2). While adjacent monomers showed 30%–45% sequenced divergence, pairwise sequence comparisons of dimeric repeats showed between 2%–5% sequence divergence (Table S5; Kimura 2 parameter). Similar values were obtained based on comparisons between the encoded pattern sets, suggesting considerable homogeneity in the structure and organization of macaque centromeric satellites (as predicted by restriction digest analysis [21].

**Figure 4:**
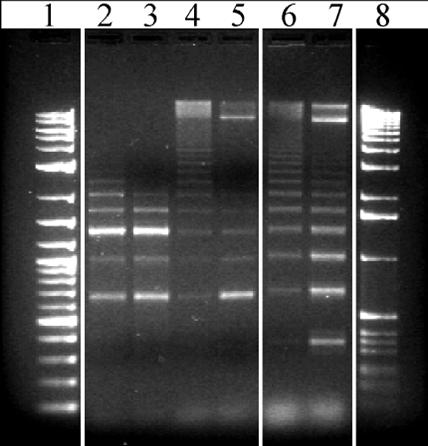
Examples of Restriction Enzymatic Digestion on Primate Fosmid Clones Containing HOR Alpha-Satellite DNA Partial and complete digestion of the fosmid chimpanzee clone CH1251-783f21 (HOR3; columns 2 and 3, respectively), chimpanzee clone CH1251-518E17 (HOR4; columns 4 and 5), and macaque fosmid clone MQAD-1143O3 (macaque-HOR; columns 6 and 7, respectively). Partial digests confirm HOR structure, while nearly complete digests confirm expected size of predominant repeat units within the array. Columns 1 and 8: log-2 DNA ladder and 1-kb ladder markers, respectively.

In contrast, the chimpanzee encoded pattern set showed considerably more diversity in structure, more reminiscent of human centromeric DNA architecture (Table 4). The average chimpanzee paired-end statistic for these pattern sets (37.21%) was similar to accurately predicted HORs in humans, predicting the presence of HORs in chimpanzees. Interestingly, the assembled chimpanzee sequences showed >12% sequence divergence when aligned to human HOR sequences (maximum sequence identity between 78%–88% between human and chimpanzee HORs; Table S3). As a test of our in silico prediction of HOR structure, we retrieved a chimpanzee fosmid clone corresponding to seven of the chimpanzee alpha-satellite HORs. We designed a specific restriction enzyme assay to digest once and only once within the chimpanzee higher-order array (not including the fosmid polylinker multiple-cloning site). Partial and complete restriction enzymatic digestions confirmed the presence of an alpha-satellite HOR structure in all subclones. In six of seven cases, the observed fragment sizes were consistent with that expected based on in silico analyses (Figure 4 and Table 3). Presence of distinct dimeric ladder-sized bands in complete digests suggests a lack of homogeneity or a more degenerate structure in chimp HOR arrays. Similarly, restriction digests of macaque fosmid clones confirmed multiples of the basic dimeric repeat pattern.

**Figure 5:**
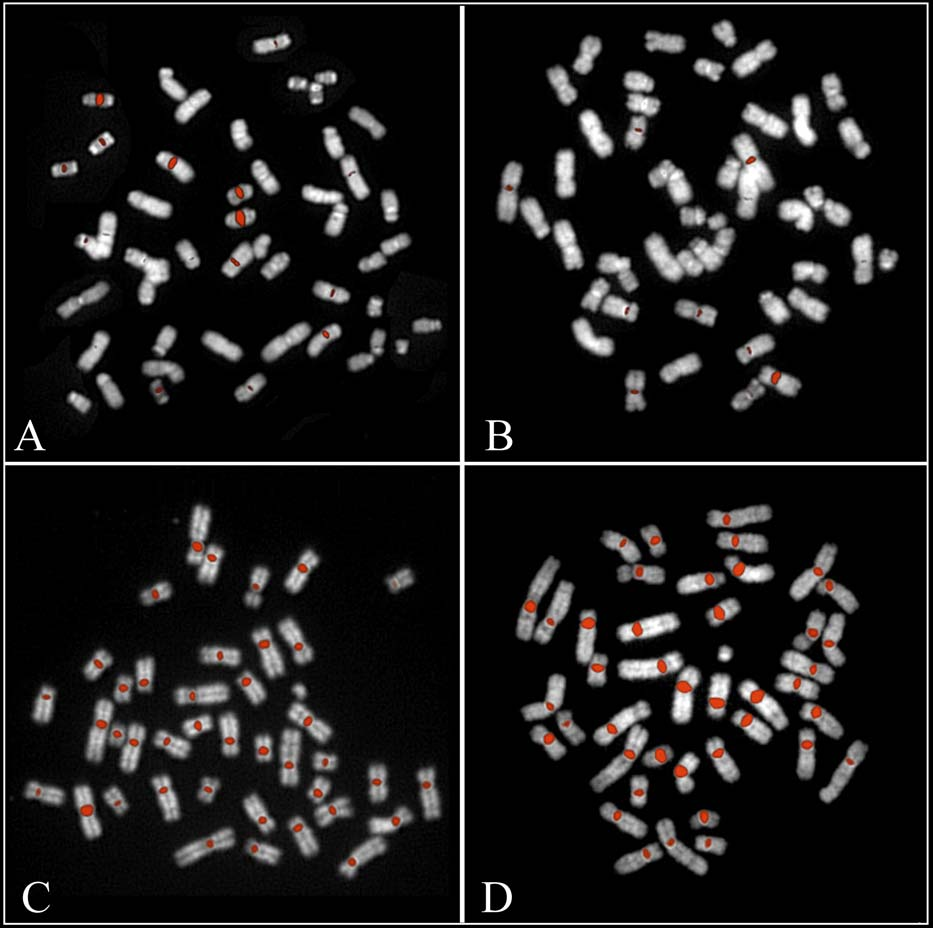
Nonhuman Primate Alpha-Satellite FISH Chimpanzee fosmid probes (A) CH1251-2018k17 and (B) CH1251-1027N15 containing putative HOR alpha-satellite repeats showed specific centromeric and pericentromeric signals when hybridized to chimpanzee chromosomes. (C) Baboon probe (RPCI-100L5) and (D) macaque BAC (CHORI250-102K3) show a pancentromeric distribution when tested against metaphases from the corresponding species. Similar results obtained for all putative HORs identified from the macaque (unpublished data). All the reported FISH experiments were performed with high stringency: three washes with 0.1× SSC at a temperature of 60 °C.

As a final test, we selected a fosmid clone representing each of the chimpanzee and macaque HOR units and assessed its chromosomal distribution by metaphase FISH analysis. In humans, it has been shown that centromeric HOR units are grouped into suprafamilies, and that subsets of nonhomologous chromosomes share monomer alpha-satellite sequences from the same suprafamily. Consequently, probes representing a specific HOR unit can cross-hybridize to centromeres from nonhomologous chromosomes under low stringency hybridization conditions. For the chimpanzee HOR, we observed each of the predicted HOR hybridizing to the centromeres of a set of nonhomologous chromosomes (Table 3 and Figure 5A and 5B). Unlike human HORs, we noted several secondary signals mapping to pericentromeric locations on chimpanzee chromosomes. Moreover, even under high-stringency conditions, a single signal to a specific chromosome was seldomly observed. As predicted [2,5–7], hybridization of the chimpanzee probes against human metaphases mapped to the centromeres and pericentromeric regions of nonorthologous chromosomes (Figure S3). We note that not all chimpanzee centromeres were identified in this analysis, indicating that only a fraction of the HORs have been successfully identified. Furthermore, some chromosomes (e.g., Chromosomes 19 and 20) were common to a large number of the probes. Interestingly, even in cases where the FISH patterns appeared virtually identical (PTRHOR 3 and PTRHOR 8), a sequence comparison revealed that the two HORs shared only 78.6% sequence identity, suggesting the presence of two different HOR units on the same chromosome. Fosmids that were used as probes were required to have end sequences matching to the same pattern matching set. We did not FISH those where one end mapped to HOR and the other did not. Such fosmid clones may represent edges of arrays with diverged alpha-satellite.

In contrast to the human and chimpanzee, each probe isolated from the macaque and baboon libraries cross-hybridized equally well to all chromosomes (with the exception of the Y chromosome; Figure 5C and 5D) [21,22]. Reciprocal experiments (where baboon probes were hybridized to macaque, and vice versa) confirmed a long-standing, predominant pancentromeric signal distribution in both species (Figure S3). Despite numerous experiments, no probe could be unambiguously assigned to a specific chromosome in these species. These data suggest fundamental differences in the structure and organization of centromeric DNA between the Old World and great ape primate lineages [2,21,22].

### Phylogenetic Analysis of Alpha-Satellite Sequences

**Figure 6:**
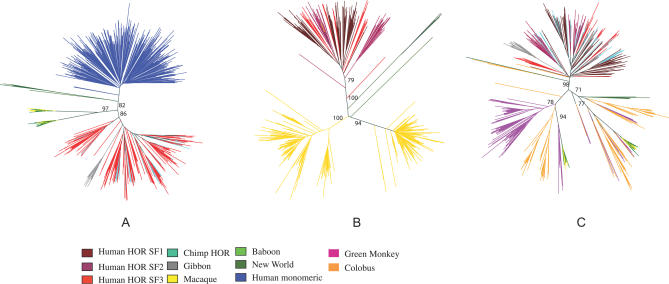
Primate Phylogenetic Analyses of Alpha-Satellite Sequences Neighbor-joining methods were used to construct (A) a phylogenetic tree of human monomeric alpha-satellite sequences from Chromosome 8 (blue); putative HOR sequences from human (red), chimp (cyan), and gibbon (gray); and random samples from macaque (yellow) and baboon (green); and (B) a phylogenetic tree comparing all human HORs versus macaque HOR sequences identified in this study; and (C) a phylogenetic tree comparing randomly ascertained alpha-satellite monomers from four different Old World monkey species. New World monkey alpha-satellite sequences (dark green) are included as an outgroup in these analyses. Bootstrap values (*n* = 100 replicates) greater than 75 are indicated on the branches.

In an effort to assess the evolutionary history of primate alpha-satellite sequence, we examined the phylogenetic relationship between both monomeric and higher-order alpha satellite sequences extracted from primate WGS sequence data. In these analyses, we included all higher-order alpha satellite consensus sequences from human, chimpanzee, and gibbon centromeric regions; dimeric alpha-satellite sequences from macaque and baboon; monomeric alpha satellite sequences from New World monkey [6]; and monomeric alpha-satellite sequence located at the periphery of Chromosome 8 [8]. In light of the large number of sequence taxa of limited length, we performed 100 bootstrap tests for each phylogenetic analysis. Our analysis reveals a tripartite evolutionary relationship among these primate sequences; Old World monkey, ape higher-order, and human monomeric alpha-satellite are each evolutionarily distinct (Figure 6). The data show clear introgression of our predicted chimpanzee HORs, with human suprafamily designations, while our limited survey of gibbon sequences suggest the possibility of a distinct origin from a common set of ape ancestral HOR sequences. The dimeric repeat structure is the fundamental unit of macaque centromeric DNA (Figure 6B). Random sampling, as well as testing of alpha-satellites mapping to encoded pattern sets from the macaque, all show a distinct bifurcation (Figure 6, Figure S4, and unpublished data). Analysis of alpha-satellite sequences identified from random BAC end sequences of the colobus, African green monkey, and baboon confirm that the dimeric repeat structure is common to all Old World monkey species (Figure 6C).

## Discussion

The current model of primate centromere DNA organization has been developed almost exclusively from FISH and restriction enzyme studies of the human genome in the last 25 years [4,5,23]. These efforts required the systematic cloning and sequencing of heterochromatic DNA, frequently from chromosome-specific reagents. Our understanding of the extent of sequence and structural diversity among nonhuman primates is much more limited [2,11,21,22,24–26]. We developed an algorithm to identify, categorize, and reconstruct HOR structures from genome-wide sequence data. In this study, we analyzed more than 1.42 Gb of sequence primarily from three species to identify 265,868 (Table 1) alpha-satellite repeat units corresponding to an estimated 100,000 BAC and fosmid clones. Our results provide a genome-wide perspective on the evolution and structure of these regions and a clone framework for further evolutionary, cytogenetic, and sequence characterization.

We have demonstrated that it is possible to reconstruct known HOR alpha-satellite organization in humans via an algorithm that exploits the multimeric tandem repeat organization and the extensive intrachromosomal sequence homogenization of alpha-satellites. Although many human HOR sequences could be identified (Tables 2 and S1), not all were recovered from analysis of WGS sequence. Although restriction enzyme and subcloning biases are most likely responsible for this, our analysis of different human genome libraries of various insert size, vector type, and subcloning strategies (including WGS from randomly sheared DNA) showed virtually identical biases (Table S1). In addition, not all of those correctly identified as human HORs could be properly assembled into a pattern set that completely corresponded to the known sequence array (Table S2). Due to these limitations, our approach should be viewed as opportunistic at this point, as opposed to comprehensive. Advances in sequencing technology that obviate the need for subcloning may lead to better characterization of centromeric DNA [27].

The most important factor in correctly predicting HOR pattern sets was the Hamming distance choice for clustering of repeats. There is a tradeoff between sensitivity and specificity. A Hamming distance estimate that is too low will fail to cluster related repeats, while increasing the value will lead to overcollapse and a concomitant loss of power to accurately distinguish HOR pattern sets. In humans, we optimally set the Hamming distance to 4 based on paralogous sequence divergence between multimeric units within the human HOR arrays. In a blind study of human WGS sequence, we estimate that approximately 12 of 24 (Table 2) multimeric units can be partially or fully reconstructed at this distance.

The heuristics described to merge pattern sets may also impose problems in HOR array prediction. If there exists two different HOR sets that include monomers of high sequence identity (<2% divergent), the pattern-merging scheme may generate chimeric higher-order structures. For this reason, we only use the HOR structures that are experimentally verified as part of our phylogenetic analysis. In addition, all the HOR structures reconstructed using human WGS reads are either identical to previously published HOR arrays, or validated experimentally. Similarly, all but one computationally predicted HOR structure in the chimpanzee can be experimentally validated.

The availability of paired-end sequence data and corresponding clone reagents provide additional tools for confirmation. Our analysis of human WGS data, for example, identified a previously undescribed HOR sequence structure (HSAHOR8) and corresponding clones for testing. Mate-pair data from human fosmid ends (40-kb inserts) confirm that 99.35% of the pairs map to the same pattern set, confirming tandem reiterations of this multimeric repeat unit. FISH analysis of a corresponding fosmid clone from the library (Figure 3) map the novel higher-order sequence to the primary constriction of Chromosomes 14 and 22. Similarly, analysis of chimpanzee fosmid paired-end sequence data identified seven novel HOR units of various lengths (Table 3), and FISH analysis assigned each of these to specific centromeres on chimpanzee chromosomes (Figure 5A and 5B).

Phylogenetic analyses confirm that human and chimpanzee HOR alpha-satellites share a common origin [23] that is evolutionarily distinct from the flanking peripheral monomeric sequences. Every major human alpha-satellite suprachromosomal family shares homologous sequences with chimpanzee (Figures 6A and S5), despite the fact that they map to nonorthologous chromosomes between the two species (Table 3). A comparison of gibbon alpha-satellites reveals only limited introgression with human–chimpanzee sequence clades. These data suggest that gibbon HORs evolved, in large part, independently from that of the human and chimpanzee. It should be noted however, that the number of gibbon sequences is significantly fewer (Table 1). In addition, the gibbon sequences are derived from a large-insert BAC library where restriction enzyme subcloning biases are thought to be more pronounced. Additional sequencing of the gibbon genome in smaller insert libraries may reveal other, yet unreported sequences and phylogenetic relationships.

Comparisons between ape and Old World monkey alpha-satellite DNA confirm two radically distinct patterns of centromeric organization and chromosome distribution [21,22,25]. Almost all (80% of all monomers at Hamming distance = 10) macaque alpha-satellite sequences are organized around a distinct dimeric repeat structure configuration (Figure 6B). Sampling of different Old World monkey species (including colobus, African green monkey, macaque, and baboon) confirm that the dimeric structure is ancient (15–20 million years old) based on the estimated evolutionary divergence of these species [28]. FISH analysis with either baboon or macaque probes reveal a pancentromeric distribution on metaphase chromosomes (testing of representative clones from each of the ten HOR pattern sets showed no difference; Figure 5). Unlike the great ape higher-order alpha-satellite, HOR structures cannot be assigned to a specific chromosome in these species. These data provide compelling evidence that intrachromosomal homogenization of alpha-satellite DNA has predominated in humans and apes, while transchromosomal exchanges have been the dominant mode among all Old World monkey species.

In summary, we have shown that we can systematically extract evolutionary data regarding centromeric DNA structure and organization from the 2%–5% of WGS sequence data that is typically excluded as part of genome sequencing projects. We provide one of the first genome-wide analyses of centromere structure and evolution from human, chimpanzee, and macaque. Fundamental differences in the structure and organization of centromere DNA between ape and Old World monkey lineages are confirmed [21,22]. The availability of these clone reagents provides a resource for further functional and sequence characterization of primate centromeres and pericentromeric transition regions [29].

## Methods

### Alpha-satellite sequences.

We constructed a nonredundant reference set of 254 monomer units from published human higher-order alpha-satellite DNA sequences [6], tracking their suprafamily designation [5,6]. We classified 188 units as canonical human HOR sequence and distinguished an additional 66 as *divergent HOR units* due to their association with atypical or more divergent centromeric arrays (e.g., Y chromosome and short arm of acrocentric chromosomes). An additional ∼270,000 alpha-satellite monomers were obtained from WGS sequences from various published primate genomic sequencing projects [14,15,30–32]. Sequence and corresponding paired-end sequence annotation was obtained from the National Institutes of Health trace repository (http://www.ncbi.nlm.nih.gov/Traces/trace.cgi) from two human library sources (Fosmid library [WIBR2 ] [31]) and WGS data from Celera [30]) and three nonhuman primate libraries, including chimp (Pan troglodytes) fosmid library (CHORI-1251) [15], rhesus macaque (Macaca mulatta) fosmid library (MQAD) [14], and Northern white-cheeked gibbon (Nomascus leucogenys) BAC genomic library (CH271). A small subsample (300–500 alpha-satellite monomers per species) was obtained from randomly end-sequenced BAC clones from various Old World monkey species, including olive baboon (Papio hamadryas anubis; RPCI-41), vervet monkey (Cercopithecus aethiops; CH252), and black-and-white colobus monkey (Colobus guereza; CH272). We would expect to recover more alpha-satellite sequences from Old World Monkey genomes. However, restriction bias limits subcloning of particular regions, especially in the case of BAC subclones. It is also the likely reason we do not recover all HOR sequences in humans. As a representative of human monomeric DNA lacking higher-order structure, we extracted (360 monomers) from a previously described genomic clone mapping peripherally of higher-order alpha-satellite DNA. We also extracted 71 monomers from another genomic clone mapping peripherally of higher-order alpha-satellite DNA on Chromosome 19 to further validate the phylogenetic relationship of monomeric versus HOR alpha-satellite sequences.

### Hierarchical clustering.

Alpha-satellite DNA sequences were retrieved from WGS data from human, chimpanzee, gibbon, and macaque fosmid and BAC end sequences used as part of genome sequencing projects (Table 1). Reads containing alpha-satellite sequences were initially identified by BLAST sequence similarity searches (*p = blastn, v = 10,000),* and individual monomer units were extracted using a customized RepeatMasker library [33] with higher-order alpha-satellite consensus sequences in [6] (parameters: -no_is –nolow –lib ‘hor.fa'). We extracted alpha-satellite monomers with the same begin and end positions based on RepeatMasker coordinates [33]. This procedure generated a total of 265,868 alpha-satellite monomer repeat units. For each species, we constructed all possible pairwise alignments for each monomer pair and computed the *aligned Hamming distance* (defined as the minimum number of substitutions required to change one string into the other) between each pair [17] (not counting indels) as follows: Hamming distance computation is solvable in *O*(*n*) time for a pair of sequences of length *n*. Here, we compute Hamming distance of pairwise alignments; thus, computation of aligned Hamming distance takes *O*(*n^2^*) time for a pair of sequences, and *O* (*k* · *m* · *n* ^2^) time for *m* repeat units against *k* alpha-satellite. To compute the aligned Hamming distance faster, we exploited the fact that the divergence of any pair of alpha-satellite sequences is less than 40%. We first built the multiple sequence alignment of all 188 sequences in the HOR set via Clustal W [34] and used the computed consensus sequence of the alignment as a centroid, where it is aligned pairwise with all WGS repeat units. This step is reminiscent of the “center-star multiple alignment” method described in [35] (pp. 348–350). If any gaps are inserted to the centroid as a result of a pairwise alignment with a WGS repeat unit *s_i_*, the bases in *s_i_* that correspond to a gap in the centroid are removed. Thus all the sequences are converted to a new version of the sequence, where all the sequences are of equal length, and the bases that can be optimally aligned to the consensus (therefore conserved in most monomers) are readjusted to the same location within the sequence. As stated above, we only count the number of substitutions during the Hamming distance computation, and indels are not penalized. Any bases inserted in a monomer but not present in the consensus (thus a specific insertion for that monomer) would induce gaps to the other monomer when pairwise alignments are performed. This method of normalizing the sequences precipitates the removal of such bases inserted in a monomer that would not be counted in any case, while aligning the conserved regions (along with substituted bases) to the same coordinates*.* This ensures that the Hamming distance of any two alignments of repeat units against the centroid would be the same as their aligned Hamming distances. The pairwise alignment of *m* repeat units and *k* higher-order consensus sequences with the centroid is completed in *O* ((*m* + *k*) · *n^2^*) time, Hamming distances for all pairs of sequences take *O* (*k* · *m* · *n*) time, and the overall distance computation time is thus reduced to *O* ((*m* + *k*) · *n* ^2^ + *k · m · n*). Once the Hamming distance was computed for each pair, we classified monomers into one of three categories: (1) repeat units that have aligned Hamming distance at most four to at least one of the consensus sequences in HOR or divergent HOR unit sets (typical divergence of monomers within an array is <2%; we therefore set the typical Hamming distance to 171 × 2% = 3.41 ≈ 4) subset have aligned Hamming distance of at most four (potential new HOR units); and (3) the remaining repeat units that fail to cluster by this threshold cutoff.

### Computational prediction of new HOR units.

In the human genome, it is usually possible to partition alpha-satellite sequence into blocks of some *k* monomers (called higher-order alpha-satellite DNA, or HOR where 4 ≤ *k* ≤ 20). Such patterns can be easily deduced from high-quality sequence using the *key string* and *colorHOR* algorithms [36,37]; however, no algorithm has been designed to predict such patterns from unassembled WGS sequence data. We developed a new algorithm, *HORdetect*, to recognize such motifs from unassembled WGS sequence by a greedy clustering method. Our primary concern at this step is to build clusters of sequences in which the divergence of any pairs of sequences is at most 2%. The following greedy algorithm ensures such a clustering scheme, although it is not perfect and can yield too many numbers of smaller clusters than the optimal number. An optimal clustering that minimizes the number of the generated clusters would be NP-Complete, and most approximation algorithms would still be unfeasible when the large number of input sequences are considered.

As input, we used the set of alpha-satellite repeat units *S* = {*s_1_, s_2_, …, s_n_*} and a distance function *d* (*s_i_, s_j_*) that returns aligned Hamming distance of two sequences *s_i_, s_j_*.

We generated as output a set of clusters *C* ={*C_1_, C_2_, …, C_m_*}, where the aligned Hamming distance of any pair of sequences in a cluster *C_k_* is at most 4. The greedy clustering method begins with assigning the first sequence in the set to the first cluster. The second sequence is compared with the initial one; if their pairwise Hamming distance is less than 4, it is added to the same cluster. Otherwise, a new cluster is created with the second sequence. This is iterated for all the remaining sequences, requiring that the divergence of any pairs of sequences within a cluster is less than the Hamming distance threshold of 4. Due to this conservative requirement, our aim is to cluster only those sequences that are part of a HOR. We opted to implement a greedy clustering algorithm in order to avoid sorting all pairwise alignment scores in memory. Any algorithm that has to precompute and store all possible pairwise Hamming distances is impractical when a large number of sequences are considered; for 135,816 sequences, such an alignment matrix would require more than 9.2 billion entries (9.2 GB if each entry is implemented as a single byte; 36.8 GB if entries are represented as integers). Furthermore, when a sequence is excluded from a cluster (Hamming distance >4 based on the centroid), that sequence is not compared with the rest of the sequences in the same cluster, thus reducing the computational time.

The algorithm can be formally described as:

1. Set *C*_1_ − {*s_i_*}, and *m* − 1.

2. *i*,2 ≤ *i* ≤ *n*:

 (a) if 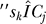 where 1 ≤ *j* ≤ *m* and *d*(*s_i_, s_k_*) ≤ 4 ; then update *C_j_* − *C_j_Ès_i_*;

 (b) otherwise, set *m* − *m* + 1 and *C_m_* − {*s_i_*}..

After the clustering step, all the clusters are assigned a number *i* = {1…*m*}. Then, corresponding WGS reads are encoded with the cluster patterns of the repeat units. For example, if a WGS read includes three monomeric repeat units from three different clusters *C_k_,C_l_,C_m_*, then that read is identified with pattern (*k*,*l*,*m*). WGS reads with the same cluster sets (patterns) are grouped together, and trivial patterns are merged; i.e., patterns (*k*,*l*,*m*) and (*l*,*m*,*t*) are collapsed to (*k*,*l*,*m*,*t*). The merging process is iterated as long as there are patterns that can be merged. In case of conflicting patterns, i.e., (*k,l,m*), (*l,m,t*), and (*l,m,z*), two separate new pattern sets are constructed as (*k,l,m,t*) and (*k,l,m,z*). A schematic representation of our alpha-satellite HOR detection algorithm can be found in Figure 1. Reads with the same pattern sets were then assembled with *phrap* [19] and *consed* [20] tools (with default parameters) to generate a consensus sequence contig (GenBank accessions). We validated the new consensus sequences computationally by examining paired-end sequences and adjacency statistics (see text).

### Phylogenetic analysis.

FASTA-formatted sequences were obtained corresponding to each of the extracted alpha-satellite monomers, and multiple sequence alignments were constructed using Clustal W (version 1.83) [34]. Due to the large number of sequence taxa, neighbor-joining methods were used to construct unrooted trees (complete deletion parameters, 100 bootstrap iterations). Phylogenetic trees were visualized using HyperTree hyperbolic tree viewer [38].

### FISH and restriction enzyme digestion.

Fosmid genomic clones corresponding to chimpanzee, human, and macaque HORs were obtained from Children's Hospital Oakland Research Institute (CHORI) or Washington University Genome Sequencing Center (WUGSC). Fosmid insert DNA was purified (1–2 μg) and digested with diagnostic restriction enzymes under partial (0.6 U/30 min) and complete restriction conditions (1 U/1 h). Primate fosmid DNAs were hybridized as FISH probes against metaphase spreads obtained by PHA-stimulated lymphocytes from normal donors and primate metaphase chromosomes (Figures 3 and 5; H. sapiens, P. troglodytes, M. mulatta, and Papio anubis) as previously described [39]. Both high- and low-stringency FISH experiments were performed using the following conditions: high stringency, three washes with 0.1× SSC at a temperature of 60 °C; low stringency, three washes with 50% formamide at 37 °C followed by three washes with 2× SSC at 42 °C. The reported FISH experiments are performed using high stringency.

## Supporting Information

Figure S1 HOR Alpha-Satellite WGS Representation versus Expected Estimated (A) minimum and (B) maximum array size correlation graphs of HOR representations in the WIBR-2 human fosmid library based on the number of aligned end sequences versus the length of the array. The *R* ^2^ values are (A) 0.1336, (B) 0.0912.
(709 KB EPS)

Figure S2 Macaque Dimeric Alpha-Satellite Repeat Structure The layout of macaque fosmid end sequences is shown over the computationally predicted dimeric array (red bars). Black lines represent the fosmid insert with corresponding forward and reverse end sequences.
(2.5 MB EPS)

Figure S3 Cross-Species FISH Experiments Chimpanzee fosmid probes (A) CH1251-2018k17 and (B) CH1251-1027N15 containing putative HOR alpha-satellite repeats showed specific centromeric signals when hybridized to human chromosomes that are nonorthologous to chimpanzee (C) Baboon probe (RPCI-100L5) and (D) macaque BAC (CHORI250-102K3) show a pancentromeric distribution when tested against metaphases from the macaque and baboon, respectively. All the reported FISH experiments were performed with high stringency: three washes with 0.1× SSC at a temperature of 60 °C.
(3.4 MB EPS)

Figure S4 Phylogenetic Analysis of Macaque Alpha-Satellite Monomers More than 85% of all macaque alpha-satellites can be clustered into pattern sets when a Hamming distance of 30 is used. Alpha-satellite monomers mapping to encoded pattern sets were sampled at each Hamming distance (but not at the next most stringent Hamming distance) and a neighbor-joining tree was constructed. A “dimeric” signal is observed phylogenetically for both the divergent and most identical pattern sets.
(2.2 MB EPS)

Figure S5 Primate Phylogenetic Analyses of Alpha-Satellite Sequences The phylogenetic tree of human monomeric alpha-satellite sequences (blue), putative HOR sequences from human (red), chimp (cyan), and gibbon (gray), and random samples from macaque (yellow) and baboon (green). This tree is similar to the one presented in Figure 6A, only the monomeric sequences from Chromosome 8 are replaced with monomeric sequences from Chromosome 19.
(1.1 MB EPS)

Table S1 Representation of Known Human HOR Satellites within WIBR2, Celera, BCM-HWW, and S213 (59 KB XLS)

Table S2 Initially Unrecognized and/or Partially Reconstructed Human Alpha-Satellite HORs (15 KB XLS)

Table S3 Human versus Chimpanzee Sequence Divergence of HORs (13 KB XLS)

Table S4 Coefficient of Determination (*R* ^2^) Values for the Expected and Actual Number of HOR Representations (13 KB XLS)

Table S5 Pairwise Kimura 2 Parameter Distances of Extracted Monomers in Macaca mulatta “HOR” (40 KB XLS)

### Accession Numbers

The GenBank (http://www.ncbi.nlm.nih.gov/GenBank) accession numbers for the structures discussed in this paper are monomeric alpha-satellite DNA on Chromosome 8 (AC026005), monomeric alpha-satellite DNA on Chromosome 19 (AC010523), higher-order repeat sequence D2Z1 (M81229), and a clone from Chromosome 22 (BX294002.19).

## Abbreviations

BAC: bacterial artificial chromosome
FISH: fluorescence in situ hybridization
HOR: higher-order repeat
WGS: whole-genome shotgun

## References

[pcbi-0030181-b001] Mahtani MM, Willard HF (1990). Pulsed-field gel analysis of alpha-satellite DNA at the human X chromosome centromere: High-frequency polymorphisms and array size estimate. Genomics.

[pcbi-0030181-b002] Warburton P, Haaf T, Gosden J, Lawson D, Willard H (1996). Characterization of a chromosome-specific chimpanzee alpha satellite subset: Evolutionary relationship to subsets on human chromosomes. Genomics.

[pcbi-0030181-b003] Warburton PE, Willard HF (1995). Interhomologue sequence variation of alpha satellite DNA from human chromosome 17: Evidence for concerted evolution along haplotypic lineages. J Mol Evol.

[pcbi-0030181-b004] Willard HF, Waye JS (1987). Chromosome-specific subsets of human alpha satellite DNA: Analysis of sequence divergence within and between chromosomal subsets and evidence for an ancestral pentameric repeat. J Mol Evol.

[pcbi-0030181-b005] Lee C, Wevrick R, Fisher RB, Ferguson-Smith MA, Lin CC (1997). Human centromeric DNAs. Hum Genet.

[pcbi-0030181-b006] Alexandrov I, Kazakov A, Tumeneva I, Shepelev V, Yurov Y (2001). Alpha-satellite DNA of primates: Old and new families. Chromosoma.

[pcbi-0030181-b007] Archidiacono N, Antonacci R, Marzella R, Finelli P, Lonoce A (1995). Comparative mapping of human alphoid sequences in great apes using fluorescence in situ hybridization. Genomics.

[pcbi-0030181-b008] Alkan C, Eichler EE, Bailey JA, Sahinalp SC, Tuzun E (2004). The role of unequal crossover in alpha-satellite DNA evolution: A computational analysis. J Comput Biol.

[pcbi-0030181-b009] Horvath J, Viggiano L, Loftus B, Adams M, Rocchi M (2000). Molecular structure and evolution of an alpha/non-alpha satellite junction at 16p11. Hum Molec Genet.

[pcbi-0030181-b010] Mashkova T, Oparina N, Alexandrov I, Zinovieva O, Marusina A (1998). Unequal cross-over is involved in human alpha satellite DNA rearrangements on a border of the satellite domain. FEBS Lett.

[pcbi-0030181-b011] Rudd MK, Wray GA, Willard HF (2006). The evolutionary dynamics of alpha-satellite. Genome Res.

[pcbi-0030181-b012] Schueler MG, Higgins AW, Rudd MK, Gustashaw K, Willard HF (2001). Genomic and genetic definition of a functional human centromere. Science.

[pcbi-0030181-b013] Consortium IS (2001). Initial sequencing and analysis of the human genome. Nature.

[pcbi-0030181-b014] Rogers J, Katze M, Bumgarner R, Gibbs RA, Weinstock GM (2002). White paper for complete sequencing of the Rhesus macaque *(Macaca mulatta)* genome.

[pcbi-0030181-b015] Consortium CSaA (2005). Initial sequence of the chimpanzee genome and comparison with the human genome. Nature.

[pcbi-0030181-b016] Needleman SB, Wunsch CD (1970). A general method applicable to the search for similarities in the amino acid sequence of two proteins. J Mol Biol.

[pcbi-0030181-b017] Hamming RW (1950). Error-detecting and error-correcting codes. Bell System Tech J.

[pcbi-0030181-b018] Warburton P, Willard H, Jackson M, Strachan T, Dover G (1996). Evolution of centromeric satellite DNA: Molecular organization within and between human and primate chromosomes.

[pcbi-0030181-b019] Ewing B, Green P (1998). Base-calling of automated sequencer traces using phred. II. Error probabilities. Genome Res.

[pcbi-0030181-b020] Gordon D, Abajian C, Green P (1998). Consed: A graphical tool for sequence finishing. Genome Res.

[pcbi-0030181-b021] Musich PR, Brown FL, Maio JJ (1980). Highly repetitive component alpha and related alphoid DNAs in man and monkeys. Chromosoma.

[pcbi-0030181-b022] Pike LM, Carlisle A, Newell C, Hong SB, Musich PR (1986). Sequence and evolution of rhesus monkey alphoid DNA. J Mol Evol.

[pcbi-0030181-b023] Alexandrov IA, Mashkova TD, Akopian TA, Medvedev LI, Kisselev LL (1991). Chromosome-specific alpha satellites: Two distinct families on human chromosome 18. Genomics.

[pcbi-0030181-b024] Schueler MG, Dunn JM, Bird CP, Ross MT, Viggiano L (2005). Progressive proximal expansion of the primate X chromosome centromere. Proc Natl Acad Sci U S A.

[pcbi-0030181-b025] Jorgensen AL, Laursen HB, Jones C, Bak AL (1992). Evolutionarily different alphoid repeat DNA on homologous chromosomes in human and chimpanzee. Proc Natl Acad Sci U S A.

[pcbi-0030181-b026] Haaf T, Willard H (1997). Chromosome-specific alpha-satellite DNA from the centromere of chimpanzee chromosome 4. Chromosoma.

[pcbi-0030181-b027] Margulies M, Egholm M, Altman WE, Attiya S, Bader JS (2005). Genome sequencing in microfabricated high-density picolitre reactors. Nature.

[pcbi-0030181-b028] Goodman M (1999). The genomic record of Humankind's evolutionary roots. Am J Hum Genet.

[pcbi-0030181-b029] Ventura M, Antonacci F, Cardone MF, Stanyon R, D'Addabbo P (2007). Evolutionary formation of new centromeres in macaque. Science.

[pcbi-0030181-b030] Venter JC, Adams MD, Myers EW, Li PW, Mural RJ (2001). The sequence of the human genome. Science.

[pcbi-0030181-b031] IHGSC (2004). Finishing the euchromatic sequence of the human genome. Nature.

[pcbi-0030181-b032] IHGSC (2001). Initial sequencing and analysis of the human genome. Nature.

[pcbi-0030181-b033] Smit AFA, Hubley R, Green P (1996–2004). RepeatMasker Open-3.0.

[pcbi-0030181-b034] Thompson JD, Higgins DG, Gibson TJ (1994). CLUSTAL W: Improving the sensitivity of progressive multiple sequence alignment through sequence weighting, position-specific gap penalties and weight matrix choice. Nucleic Acids Res.

[pcbi-0030181-b035] Gusfield D (1997). Algorithms on strings, trees, and sequences: Computer science and computational biology.

[pcbi-0030181-b036] Paar V, Pavin N, Rosandic M, Gluncic M, Basar I (2005). ColorHOR—Novel graphical algorithm for fast scan of alpha satellite higher-order repeats and HOR annotation for GenBank sequence of human genome. Bioinformatics.

[pcbi-0030181-b037] Rosandic M, Paar V, Basar I (2003). Key-string segmentation algorithm and higher-order repeat 16mer (54 copies) in human alpha satellite DNA in chromosome 7. J Theor Biol.

[pcbi-0030181-b038] Bingham J, Sudarsanam S (2000). Visualizing large hierarchical clusters in hyperbolic space. Bioinformatics.

[pcbi-0030181-b039] Barch MJ, Lawce HJ, Arsham MS (1991). The ACT cytogenetsics laboratory manual.

