# Supplementary figures and images for: Organization and Evolution of Primate Centromeric DNA from Whole-Genome Shotgun Sequence Data
Source: PLoS Comput Biol. 2007 Sep 28;3(9):e181. doi: 10.1371/journal.pcbi.0030181 (PMC1994983; doi:10.1371/journal.pcbi.0030181)

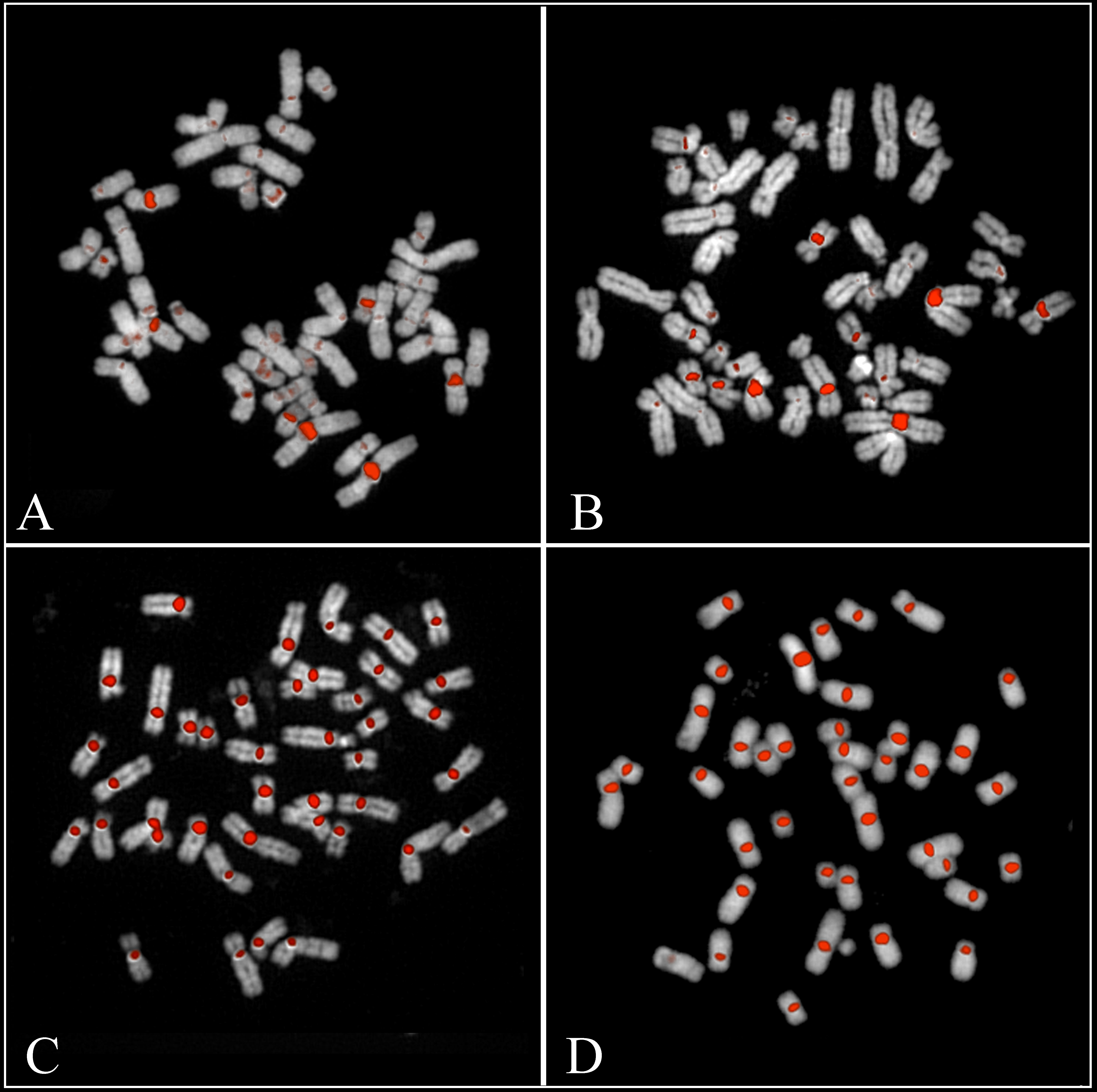

Supplement: Figure S3 — Chimpanzee fosmid probes (A) CH1251-2018k17 and (B) CH1251-1027N15 containing putative HOR alpha-satellite repeats showed specific centromeric signals when hybridized to human chromosomes that are nonorthologous to chimpanzee (C) Baboon probe (RPCI-100L5) and (D) macaque BAC (CHORI250-102K3) show a pancentromeric distribution when tested against metaphases from the macaque and baboon, respectively. All the reported FISH experiments were performed with high stringency: three washes with 0.1× SSC at a temperature of 60 °C. (3.4 MB EPS) [file pcbi.0030181.sg003.jpg]
